# High-Dose 8 mg Aflibercept for Neovascular Age-Related Macular Degeneration: Who Is Being Treated with This New Agent?

**DOI:** 10.3390/life15111657

**Published:** 2025-10-23

**Authors:** Caspar Liesenhoff, Carolin Meyrl, Daniel Krause, Franziska Eckardt, Anna Lorger, Viktoria Deiters, Johannes Schiefelbein, Julian Elias Klaas, Benedikt Schworm, Siegfried G. Priglinger, Jakob Siedlecki

**Affiliations:** Department of Ophthalmology, Ludwig-Maximilians-University, Mathildenstrasse 8, 80336 Munich, Germany; caspar.liesenhoff@med.uni-muenchen.de (C.L.); carolinmeyrl06@gmail.com (C.M.); daniel.krause@med.uni-muenchen.de (D.K.); franziska.eckardt@med.uni-muenchen.de (F.E.); anna.lorger@med.uni-muenchen.de (A.L.); viktoria.deiters@med.uni-muenchen.de (V.D.); johannes.schiefelbein@med.uni-muenchen.de (J.S.); julian.klaas@med.uni-muenchen.de (J.E.K.); benedikt.schworm@med.uni-muenchen.de (B.S.); siegfried.priglinger@med.uni-muenchen.de (S.G.P.)

**Keywords:** neovascular age-related macular degeneration, intravitreal injection, eylea 8 mg

## Abstract

**Purpose:** To describe the indication spectrum for high-dose 8 mg aflibercept for neovascular age-related macular degeneration (nAMD) in a real-world cohort in a tertiary referral center. **Methods:** The database of the University Eye Hospital Munich, Ludwig Maximilians-University was screened for eyes with nAMD treated with 8 mg aflibercept. Demographic data, multimodal imaging and treatment parameters were recorded. Reasons for treatment with 8 mg aflibercept were analyzed. **Results:** Thirty-four consecutive eyes of 31 patients (mean age 78.6 ± 8.9 years) were identified. There were 22 women (70.1%) and 9 men (29.9%). In all eyes (100%), 8 mg Aflibercept was applied as switching therapy. Prior to switching, the mean anti-vascular endothelial growth factor (VEGF) treatment duration for nAMD was 3.9 ± 2.9 years, pretreatment amounted to a mean of 34.5 ± 26.3 injections, equaling 9.2 ± 2.4 injections/year, and the mean visual acuity (VA) was 0.4 ± 0.4 logMAR. The last treatment before switching was 2 mg aflibercept in 76%, faricimab in 18%, ranibizumab in 3% and bevacizumab in 3% of cases. Reasons for switching included (A) recalcitrant nAMD with persistent fluid despite q4w dosing (17 eyes, 50%), (B) the wish for interval extension (15 eyes, 44%) and (C) macular hemorrhage (2 eyes, 6%). In group B, two-thirds of eyes (10/15, 66.7%) were maintained at ≤q6w prior to switching. **Conclusions:** In this study, high-dose 8 mg aflibercept was exclusively used as a switch therapy. Most eyes (76%) switched were from pretreatment with 2 mg aflibercept. The main reasons for switching were recalcitrant nAMD with persistent fluid despite q4w dosing (50%) or the wish for treatment extension beyond 6 weeks (32%). In the future, these data will aid in the design of prospective real-world studies comparing the efficacy of high-dose 8 mg aflibercept with older generation treatment options, especially 2 mg aflibercept.

## 1. Introduction

Treatment of neovascular age-related macular degeneration (nAMD) mainly consists of the treatment of pathological angiogenesis in the retina due to macular neovascularization (MNV) [1]. To inhibit MNV growth and exudation and thus preserve retinal anatomy, the intravitreal inhibition of anti-vascular endothelial growth factor (VEGF) has been established as a very effective and safe therapy, revolutionizing the way we view neovascular retinal disease nowadays [2].

Due to the chronicity of nAMD, which greatly outlasts the half-life of current anti-VEGF agents, patients have to routinely undergo retreatment, amounting to up to 24 injections in the first two years in the case of fixed monthly treatment [2]. To decrease treatment burden and owing to the fact that MNV reactivation represents a highly individual dynamic, the treat-and-extend regimen was introduced [3]. In the treat-and-extend regimen, patients receive an anti-VEGF loading dose of three injections, after which treatment intervals are extended by 2 weeks up to 12, 16 or 20 weeks in the case of a quiescent MNV, or shortened by 2 weeks upon MNV reactivation [4].

The treat-and-extend regimen has been widely adopted worldwide as an ideal form of individualized treatment in which MNV is treated proactively, but treatment intervals are extended to the longest interval possible without disease reactivation. Nevertheless, the treat-and-extend regimen still requires 7 to 10 injections per year, still representing a relevant burden for patients and healthcare providers alike [4].

Therefore, recent pharmaceutical efforts have focused on new intravitreal strategies with a longer intravitreal durability. These include the extension of pharmacological targets beyond VEGF, and, secondly, increases in the molar dose of an anti-VEGF agent applied. While the first strategy became available in clinics in 2022 with faricimab, a bispecific antibody inhibiting both VEFG and Angiopoietin-2 [5], the FDA and EMA approval of 8 mg aflibercept (four times the molar dose of the traditional 2 mg aflibercept) marks the most recent development in the space of intravitreal therapy of nAMD [6].

Currently, it is unclear which nAMD patients are most suited for treatment with high-dose 8 mg aflibercept.

Therefore, the aim of this study was to characterize the spectrum of indications for high-dose 8 mg aflibercept in a real-world tertiary referral center. While pivotal trials such as PULSAR [7] and CANDELA [8] have provided controlled clinical trial data on 8 mg aflibercept, evidence from routine clinical practice remains sparse. This study adds novelty by providing one of the first real-world analyses of patient selection and treatment indications for aflibercept 8 mg, thereby complementing the existing randomized trial data with practical insights relevant to daily clinical decision-making. Importantly, since pivotal clinical trials were predominantly conducted in treatment-naïve populations, our findings in a switch cohort may help to inform the design of future prospective studies by identifying which patient subgroups, particularly in tertiary referral centers, are most suitable for inclusion.

## 2. Methods

### 2.1. Participants

For this retrospective cohort study, the Smart Eye Database of the Ludwig Maximilians-University Munich, Germany, was screened for patients treated with high-dose 8 mg aflibercept between 6 March 2024 and 10 June 2024. Approval for this retrospective chart review was obtained from the ethics committee of the medical faculty of the Ludwig Maximilian’s University Munich (identifier 24-0433), and the study adhered to the tenets of the Declaration of Helsinki. As this was a retrospective analysis, the requirement for specific informed consent for study participation was waived by the ethics committee; however, written informed consent had been obtained from all patients prior to the intravitreal injection as part of standard clinical care.

Epidemiological data was obtained from each patient, including age, gender, previous ocular comorbidities and procedures, date of first diagnosis of nAMD, date of first anti-VEGF injection, type of agent used, number of total injections, date of switching to 8 mg aflibercept and objective refraction-based Snellen chart visual acuity at switching, which was later converted to logMAR for analysis. Ocular multimodal imaging was analyzed as described below.

### 2.2. Multimodal Imaging

Multimodal imaging (all on Spectralis HRA + OCT, Heidelberg Engineering, Heidelberg, Germany) was performed after pupil dilation with topical tropicamide 1% and phenylephrine 2.5% as described previously [9]. It included spectral domain optical coherence tomography (SD-OCT) and near-infrared (NIR) confocal laser scanning ophthalmoscopy (CSLO) in every eye at each visit. Fluorescein angiography (FA) was performed at baseline prior to start of treatment. Blue-autofluorescence (BAF) CSLO, indocyanine green angiography (ICG) and additional OCT angiography scans were performed at the investigator’s discretion.

### 2.3. Measurement of Central Subfield Thickness (CST)

Automated CST measurements were directly extracted from the Heidelberg Eye Explorer (Heidelberg Engineering, Heidelberg, Germany). Where needed, segmentation was manually adjusted.

### 2.4. Anti-VEGF Treatment

Prior to switching to 8 mg aflibercept, all eyes were treated using a treat-and-extend regimen with ranibizumab (Novartis Pharma AG, Basel, Switzerland), 2 mg aflibercept (Bayer, Berlin, Germany), faricimab (Roche Pharma AG, Grenzach-Wyhlen, Germany), or bevacizumab (Roche Pharma AG, Grenzach-Wyhlen, Germany). All those agents were applied in 0.05 mL. High-dose 8 mg aflibercept was applied in 0.07 mL according to the manufacturer’s guidance.

### 2.5. Statistical Analysis

Analyses were performed as described previously [9]. All data were gathered and analyzed in Microsoft Excel spreadsheets (Version 16.65 for Mac; Microsoft, Redmond, WA, USA). Statistical analysis was performed in SPSS Statistics 26 (IBM Germany GmbH, Ehningen, Germany). The level to indicate statistical significance was defined as *p* < 0.05. The Shapiro–Wilk and Kolmogorov–Smirnov tests were employed to test for normal distribution. Statistical analyses of intra-group differences were performed using the dependent two-tailed Student *t*-test and the Wilcoxon signed rank test. A repeated measures ANOVA test was used to compensate for multiple testing, if applicable. Pearson’s correlation coefficient was used to test associations between dependent and independent variables.

## 3. Results

In total, 34 consecutive eyes of 31 patients were included in this study. Baseline characteristics can be found in Table 1. The mean age was 78.6 ± 8.9 years. There were 13 right (38.2%) and 21 left (61.8%) eyes. There were 22 women (64.7%) and 9 men (35.3%). All eyes were treated with 8 mg aflibercept as a switch in anti-VEGF therapy. The mean visual acuity was 0.4 ± 0.4 logMAR at switching.

### 3.1. Reasons for Switching to High-Dose 8 mg Aflibercept

Reasons for switching are listed in Table 2. Three reasons for switching to high-dose 8 mg aflibercept were identified. In 17 eyes (50%, group A), switching was performed due to recalcitrant nAMD, defined as persistent intraretinal, subretinal or mixed intra-/subretinal fluid in spite of q4w dosing. In 15 eyes (44%, group B), switching to high-dose 8 mg aflibercept was performed with the wish to potentially extend treatment intervals; of those 15 eyes, 3 eyes (20%) were maintained at q5w, 8 eyes (53.3%) were maintained at q6w, 3 eyes (20%) were maintained at q8w 1 one eye (6.7%) was maintained q12w prior to switching. In two eyes (6%, group C), switching to high-dose 8 mg aflibercept was performed due to acute macular hemorrhage in patients of old age, which made surgical intervention and postoperative prone positioning impossible.

### 3.2. Pretreatment Characteristics

On average, anti-VEGF treatment was initiated 3.9 ± 2.9 years prior to switching. During this period, patients received a mean of 34.5 ± 26.3 anti-VEGF injections, equaling 9.2 ± 2.4 injections per year. In the last year prior to switching, injection load was similarly continuously high (8.9 ± 2.0 injections; *p* = 0.141).

The last treatment before switching was 2 mg aflibercept in 26 eyes (76%), faricimab in 6 eyes (18%), ranibizumab in 1 eye (3%) and bevacizumab in 1 eye (3%).

### 3.3. Macular Neovascularization (MNV) and Fluid Characteristics

**Figure 1:**
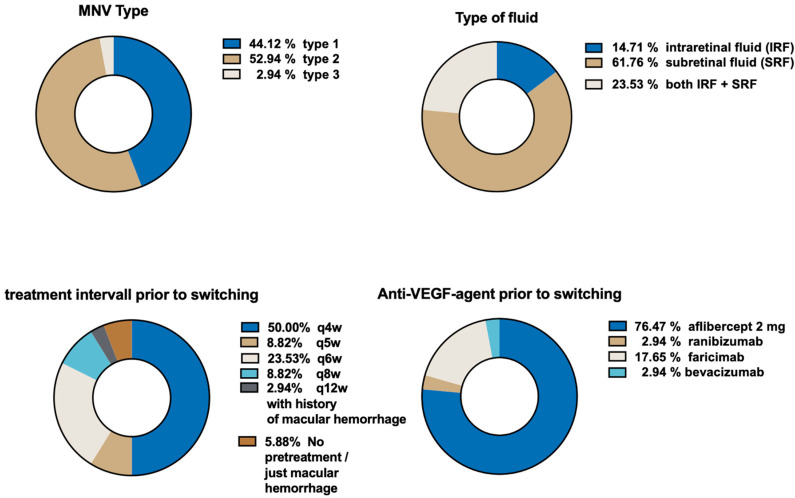
Pretreatment and disease characteristics.

MNV was type 1 in 15 eyes (44%), type 2 in 18 eyes (53%) and type 3 in 1 eye (3%) (Figure 1). Mean CST at switch was 420.1 ± 135.7 µm. Prior to switching, 5 eyes (14.0%) had IRF, 21 eyes (62.0%) had SRF and 8 eyes (24.0%) had both IRF and SRF. Two eyes had macular hemorrhage (6%) which, due to the advanced age of the patients and difficult surgical circumstances, were only treated with 8 mg aflibercept.

## 4. Discussion

This study is the first to describe the real-world indication spectrum of high-dose 8 mg aflibercept in patients with nAMD. Notably, all eyes treated with 8 mg aflibercept in the study period represented switch patients, which is not unusual in the early phase after the approval of a new drug. Our observations indicate that, in clinical practice, switching to aflibercept 8 mg predominantly occurred when there was persisting sub- or intraretinal fluid despite intensive anti-VEGF treatment, primarily administered in q4w to q6w intervals. As an important finding, 76% of eyes were switched to 8 mg aflibercept from 2 mg aflibercept, 50% of eyes were switched due to recalcitrant nAMD with persistent fluid in spite of q4w dosing and 44% of eyes were switched from q5w and q6w intervals with the wish of treatment interval extension with the higher aflibercept dose. These observations align with the fact that, in our studied switch cohort, there was a trend to an increase in the prevalence of type 2 and 3 MNV (56%) compared to similar studies reported in the literature, which documented prevalences ranging from 42.3% to 61.3% [7,10,11].

While not surprising or unexpected, these real-world data offered in our study represent an important framework for future prospective randomized trials. Depending on a multitude of factors like the intravitreal potency to suppress exudation, durability, or safety (considering the recent cases of intraocular inflammation with brolucizumab [12,13,14,15,16]), distinct indication spectrums for novel intravitreal agents for the treatment of nAMD might exist. For example, a multitude of studies on switching treatment to faricimab for nAMD have been published, but they very much differ in design and inclusion criteria, which makes direct comparisons complicated [17,18,19,20,21]. Therefore, based on real-life data offered in our study, clinicians and researchers can identify who will most likely be deemed suitable for a switch to high-dose 8 mg aflibercept, and, even more importantly, which agents will most likely have been used for pretreatment (to compare against). These data can then inform the design of prospective studies, and, optimally, randomized cross-over trials that can prove or refute potential benefits of increasing the dose of aflibercept from 2 to 8 mg.

Varying opinions exist on whether the efficacy and durability of aflibercept can be greatly improved by increasing the molar dose by four times. In the Phase II CANDELA trial, 8 mg aflibercept did not surpass the efficacy of 2 mg, but trends towards an anatomical benefit could be observed [8]. In the recent Phase III PULSAR trial, the efficacy of 8 mg aflibercept was non-inferior to 2 mg while durability was stated to be extended substantially [7]. Unfortunately, PULSAR had retreatment criteria which did not only, as usual, rely on anatomical features, i.e., fluid on OCT, but also required patients to simultaneously lose ≥5 ETDRS letters in BCVA to have retreatment [7]. As OCT-guided retreatment represents the standard of care nowadays [22], these slightly artificial retreatment criteria including a defined loss of function do not allow for final evidence on treatment durability compared to standard 2 mg aflibercept.

It might therefore be helpful to look at evidence from other historic trials investigating increases in the molar dose of anti-VEGF agents. In the HARBOR trial investigating 2.0 mg ranibizumab (four times standard dose of 0.5 mg), superiority of 2.0 mg vs. 0.5 mg concerning BCVA could not be demonstrated [23]. On the other hand, HARBOR was powered for superiority concerning visual acuity, while nowadays most clinical trials aim at non-inferiority, but with fewer injections [5,10]. The latter goal was accomplished by the HAWK and HARRIER trials which reported the efficacy of brolucizumab, a novel single chain antibody fragment that definitely differs from ranibizumab and aflibercept—but mostly by the smaller size of the molecule itself, which allowed to increase the dose applied intravitreally to 6.0 mg, and represented the first substance to achieve q12w dosing [10]. Accordingly, faricimab, a bispecific antibody against VEGF and Angiopoietin-2, investigated in TENAYA and LUCERNE, is also applied at a higher dose of 6.0 mg, allowing for q12w and q16w dosing [5].

Concerning aflibercept, some smaller studies investigating more frequent dosing than the usual q8w or increasing the molar dose (both off-label) can be found in the literature. A study published by You et al. in 2018 [24], for example, treated cases of recalcitrant nAMD with 4 mg aflibercept (double-dose, off-label) q4w and found sustainable significant anatomical improvement after one month, and, rare for switch studies, improvements in visual acuity of at least one line in 45% of the treated eyes. Similar improved outcomes were reported with 2 and 3 mg aflibercept by Nielsen et al. [25] and 3 mg aflibercept bei Feng et al. [26]. In a comparative study, Broadhead et al. investigated 3 mg aflibercept (1.5-fold dose), 1.8 and 2.5 mg bevacizumab (1.5-fold and 2.0-fold dose) and 0.75 and 1.0 mg ranibizumab (1.5-fold and 2.0-fold dose) and found improved efficacy with increased dosing for all agents examined. Therefore, improved anatomical and functional outcomes, along with enhanced durability, might be feasible. In this context, comparative trials between 2 and 8 mg aflibercept should be conducted. This is especially true and important for patients with recalcitrant nAMD with suboptimal response despite q4w dosing, as here the highest positive influence on visual acuity can be expected.

In conclusion, three main bullet points for the future prospective examination of high-dose 8 mg aflibercept can be established. (I) In the early adoption phase in real life, most patients receiving 8 mg aflibercept will represent switch patients. (II) The majority of eyes, in our study 76%, will be switched to 8 mg aflibercept from 2.0 mg aflibercept. (III) The majority of patients switched to 8 mg aflibercept will represent cases of recalcitrant nAMD with persistent fluid in spite of q4 dosing, or patients with q5w and q6w dosing who desire the option of extending treatment intervals. Larger cohorts from secondary and tertiary referral centers as well different geographies will further elucidate the indication spectrum for this novel intravitreal agent. These data are pivotal to design future comparative trials to gauge the efficacy of 8 mg aflibercept against other older and newer anti-VEGF agents.

Future prospective studies will be essential to determine whether the increased molar dose of aflibercept translates into superior durability and functional outcomes compared with established agents such as 2 mg aflibercept or faricimab. Moreover, ongoing post-marketing surveillance will be required to carefully assess the long-term safety profile of 8 mg aflibercept. Ultimately, the integration of high-dose aflibercept into individualized treatment algorithms may reduce injection burden, improve patient adherence and help to optimize healthcare resource utilization.

This study has several limitations. First, its retrospective, single-center design and relatively small sample size limit the generalizability of our findings. Second, the follow-up period was short and functional outcomes beyond baseline visual acuity were not systematically analyzed, precluding definitive conclusions on the long-term efficacy of aflibercept 8 mg. Finally, as this was an early adoption phase, all included patients represented switch cases, and thus the results may not be extrapolated to treatment-naïve populations. These limitations highlight the need for larger, prospective, multicenter studies with longer follow-ups to confirm and extend our observations.

## Figures and Tables

**Table 1:** Baseline characteristics.

**Parameter**	
**Eyes (n)**	**34**
**Right/Left**	**13/21**
**Patients (n)**	**31**
**Female/Male**	**22/9**
**Mean age (years)**	**78.6 ± 8.9**
**Mean injections before switching**	
–total	**34.5 ± 26.3**
–per year	**8.0 ± 2.0**
**Last treatment before switching**	
–Ranibizumab	**1 (3%)**
–Aflibercept 2 mg	**26 (76%)**
–Faricimab	**6 (18%)**
–Bevacizumab	**1 (3%)**
**Mean injection interval (weeks) at switch**	**5.2 ± 2.0**
**Mean CST (µm) at switch**	**420 ± 136**
**Mean visual acuity (logMAR) at switch**	**0.4 ± 0.4**

**Table 2:** Reasons for switching.

**Reasons for swtiching**	
**recalcitrant nAMD despite q4w**	
q4w	**17 (50.0%)**
**wish to extend treatment interval above**	**15 (44%)**
q5w	3 (20.0%)
q6w	8 (53.3%)
q8w	3 (20.0%)
q12w	1 (6.7%)
**macular hemorrhage**	**2 (6%)**

## Data Availability

The data presented in this study are available on request from the corresponding author due to legal reasons.
